# Roles of inflammasomes in viral myocarditis

**DOI:** 10.3389/fcimb.2023.1149911

**Published:** 2023-05-15

**Authors:** Jingyu Xu, Zihao Zhou, Yidan Zheng, Sai Yang, Kun Huang, Huili Li

**Affiliations:** ^1^ Department of Cardiovascular Surgery, Union Hospital, Tongji Medical College, Huazhong University of Science and Technology, Wuhan, China; ^2^ Department of Gastrointestinal Surgery, Union Hospital, Tongji Medical College, Huazhong University of Science and Technology, Wuhan, China; ^3^ Institution of Cardiology, Union Hospital, Tongji Medical College, Huazhong University of Science and Technology, Wuhan, China

**Keywords:** viral myocarditis, inflammasome, infection, cytokines, interleukin-1β

## Abstract

Viral myocarditis (VMC), characterized by viral infection-induced inflammation, is a life-threatening disease associated with dilated cardiomyopathy or heart failure. Innate immunity plays a crucial role in the progression of inflammation, in which inflammasomes provide a platform for the secretion of cytokines and mediate pyroptosis. Inflammasomes are rising stars gaining increasing attention. The nucleotide oligomerization domain-, leucine-rich repeat-, and pyrin domain-containing protein 3 (NLRP3) inflammasome, the caspase recruitment domain-containing protein 8 (CARD8) inflammasome, and the caspase-11 inflammasome are three inflammasomes that were reported to affect the process and prognosis of VMC. These inflammasomes can be activated by a wide range of cellular events. Accumulating evidence has suggested that inflammasomes are involved in different stages of VMC, including the trigger and progression of myocardial injury and remodeling after infection. In this review, we summarized the pathways involving inflammasomes in VMC and discussed the potential therapies targeting inflammasomes and related pathways.

## 1 Introduction

Myocarditis is the inflammation and injury of the myocardium resulting from multiple infectious or non-infectious factors including viruses, immune system activation, or exposure to toxins/drugs. Viruses are the main infectious cause of myocarditis, and increasing evidence proves that excessive host immune responses probably play a more crucial part in the pathogenic process of VMC (Reyes and Lerner, 1985; Mason, 2003; Fairweather and Rose, 2007; Basso, 2022). In some patients with VMC, the developing or persistent myocardial injury can lead to dilated cardiomyopathy and even heart failure (Sagar et al., 2012). However, the pathogenic mechanisms of VMC have not been well demonstrated.

Inflammasomes are multimolecular protein complexes assembling in response to pathogen-associated molecular patterns (PAMPs) or damage/danger-associated molecular patterns (DAMPs), resulting in the maturation and release of interleukin-1β (IL-1β) and IL-18 and an inflammatory form of cell death named pyroptosis (Rathinam et al., 2012; Latz et al., 2013; Sharma and Kanneganti, 2021). The IL-1β and IL-18 are widely involved in inflammatory responses and myocardial injury.

In this review, we summarized relevant literature involving the roles of inflammasomes in different pathogenic stages of VMC. Besides, we discussed the possible roles of inflammasomes in COVID-19-related myocarditis. Furthermore, we proposed potential therapies targeting the NLRP3 inflammasome-IL-1β axis.

## 2 Pathophysiology of viral myocarditis

VMC is the main subtype of infectious myocarditis and a significant cause of dilated cardiomyopathy (DCM) (Cooper, 2009; Pollack et al., 2015; Basso, 2022). VMC can be induced by a variety of viruses, including coxsackievirus B3 (CVB3), encephalomyocarditis virus (EMCV), human immunodeficiency virus (HIV), human parvovirus B 19 (PVB-19) and angiotensin-converting enzyme 2-tropic cardiotoxic viruses such as severe acute respiratory syndrome coronavirus 2 (SARS-CoV-2) (Tschöpe et al., 2021). Laboratory VMC mouse models are commonly induced by CVB3 and EMCV. Moreover, COVID-19-related myocarditis has aroused increasing attention (Siripanthong et al., 2020; Chen et al., 2020; Inciardi et al., 2020; Long et al., 2020). Although the causative virus varies, the development of VMC is manifested as an infectious phase and a post-infectious phase.

### 2.1 Infectious phase

The infectious phase is characterized by viral infection and subsequent direct damage to myocardium, including myocardial inflammation deriving from innate and adaptive immune reactions, necrosis, and apoptosis of cardiomyocytes.

The existing understanding of the pathogenic process of VMC is mainly derived from the CVB3-induced VMC mouse model. Coxsackieviruses enter cells through the combined effect of coxsackievirus-adenovirus receptor (CAR) and decay-accelerating factor (DAF) (Shenoy-Scaria et al., 1992; He et al., 2001), and subsequently affect cell function through a variety of viral proteases. Among the viral proteases, the roles of proteases 2A and 3C in cleaving cellular proteins stand out, which exacerbate the VMC (Liu and Mason, 2001; Xiong et al., 2007). The viral infection eventually leads to apoptosis and necrosis of the cardiomyocytes, increasing the release of viruses that infect the rest of the cardiomyocytes and causing damage to the myocardium.

Immune responses after viral infection play key roles in the development of VMC. On the one hand, the immune responses are prerequisites for the removal of pathogens; on the other hand, sustained and excessive immune responses may cause myocardial damage and even DCM (Sagar et al., 2012). In the context of VMC, viruses trigger innate immune responses by interacting with a variety of pathways involving melanoma differentiation-associated gene 5 (MDA5), toll-like receptors (TLRs), etc. MDA5 is essential for the production of maximal levels of interferon (IFN)-α in the early stage of infection. The absence of MDA5 inhibits the type I IFN production and exacerbates mortality in mice with CVB3-induced VMC (Wang et al., 2010; Hühn et al., 2010). Macrophages, neutrophils, dendritic cells, and other cells can recognize viruses through TLRs (Cooper, 2009; Rivadeneyra et al., 2018) and activate nuclear transcription factors such as NF-κB, promoting the production of proinflammatory cytokines including tumor necrosis factor (TNF), IL-1α, IL-1β, IL-2 and IFN-γ (Pollack et al., 2015; Lasrado and Reddy, 2020). This process involves the activation of inflammasomes such as the NLRP3 inflammasome. Furthermore, there is critical cooperation between the MDA5-mediated pathway and the TLR-mediated pathway on some occasions, such as the RIG-I/MDA5-type I IFN pathway and the TLR3-type II IFN pathway for efficient innate antiviral immune responses (Negishi et al., 2008). After the activation of innate immune responses, adaptive immune responses set off, initiating the activation and expansion of T cells and B cells and participating in the progress of VMC. The immune responses may promote cell necrosis, cardiac fibrosis, and remodeling, which can further result in severe arrhythmia, left ventricle dilation, and even heart failure (Cooper, 2009).

In EMCV-induced VMC, there is not much literature about viral internalization, but recently, a disintegrin and metalloproteinase 9 domain (ADAM9) has been identified as a major EMCV dependency factor (Bazzone et al., 2019). In the pathogenic process of EMCV-induced VMC, the viral protein 2B is crucial. The protein 2B activates the NLRP3 inflammasome by promoting calcium (Ca^2+^) flux from the Golgi apparatus and endoplasmic reticulum (ER) into the cytoplasm, along with K^+^ efflux out of the cytoplasm (De Jong et al., 2008). Inflammatory responses, including high levels of proinflammatory cytokines in the heart, can be detected in the early stage of EMCV-induced VMC and enhance the toxicity of EMCV, further impairing cardiac function (Shioi et al., 1996; Iwasaki et al., 1999; Matsumori et al., 2004). SARS-CoV-2 enters cardiomyocytes by binding its spike protein to angiotensin-converting enzyme 2 (ACE2) with the aid of transmembrane serine protease 2 (TMPRSS2) (Hoffmann et al., 2020), possibly providing the premise for the NLRP3 inflammasome activation and leading to subsequent inflammation and injury.

### 2.2 Post-infectious phase

The main features of the post-infectious phase, taking CVB3 as an example, include cardiac remodeling, cardiac fibrosis, and cardiac dysfunctions. After the infectious phase, some patients will experience remission, including a reduction of viral titers, amelioration of inflammation, and complete resolution of myocardial damage. But some other patients may experience chronic inflammation and dilated cardiomyopathy due to the persistence of the viral genome or cross-reactive antibodies (such as antibodies targeting cardiac myosin) (Fairweather et al., 1998; Calabrese and Thiene, 2003). If inflammation persists, the release of proinflammatory cytokines can lead to the activation of matrix metalloproteinases (MMPs) and the production of pro-fibrotic cytokines, both of which can cause cardiac remodeling and fibrosis. Meanwhile, antigenic cross-reactions can also aggravate cell damage (Sagar et al., 2012).

## 3 Inflammasomes

**Figure 1:**
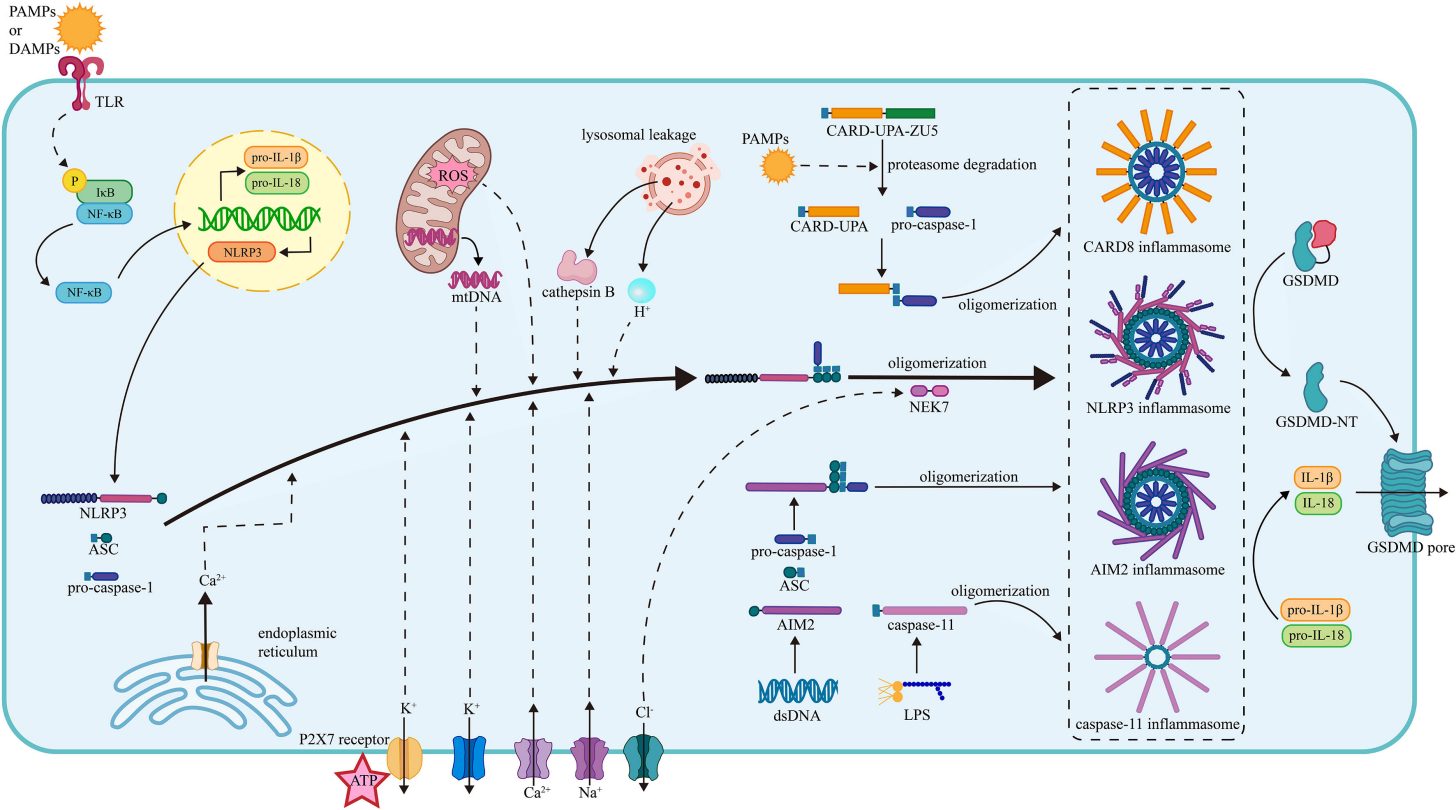
The mechanism of inflammasome activation. (1) The NLRP3 inflammasome activation. The activation of the NLRP3 inflammasome requires two steps of stimuli. The first signals are mainly PAMPs or DAMPs, interacting with the PRRs and activating the NF-κB pathway, thus promoting the transcription of NLRP3, pro-IL-1β and pro-IL-18. The second signals promote the assembly and oligomerization of the NLRP3 inflammasome. The efflux of K^+^, influx of Na^+^, mobilization of Ca^2+^ (from ER and extracellular), mtROS, mtDNA, lysosomal leakage of H^+^ and cathepsin B facilitate the assembly of NLRP3, ASC, and pro-caspase-1. The efflux of Cl^-^ facilitates the NEK7 attachment and thus the oligomerization. (2) The CARD8 inflammasome activation. The PAMPs induce the degradation of CARD8 sensor in proteasome and facilitate the release of CARD-UPA domain, which recruits pro-caspase-1 and results in the formation of CARD8 inflammasome. (3) The caspase-11 inflammasome activation. Under the stimulation of LPS, the caspase-11 inflammasome is formed, gains proteolytic activity, and cleave. (4) The AIM2 inflammasome. The AIM2 senses the dsDNA and then recruits the ASC and pro-caspase-1, facilitating the assembly and oligomerization of the AIM2 inflammasome. The oligomerization of pro-caspase-1 facilitates the maturation of caspase-1. Caspase-1 cleaves pro-IL-1β and pro-IL-18 into mature IL-1β and IL-18. Besides, the caspase-1 splits GSDMD into two fragments and the N-terminal fragment form pores on plasma membrane promoting the release of cytokines. NLRP3, nucleotide oligomerization domain (NOD)-, leucine-rich repeat (LRR)- and pyrin domain (PYD)-containing protein3; PAMP, pathogen- associated molecular patterns; DAMP, damage-associated molecular patterns; TLR, toll-like receptor; NF-κB, nuclear factor κ-light-chain enhancer of activated B cells; ASC, apoptosis-associated speck-like protein containing a CARD; NEK7, NIMA-related kinase 7; NIMA, never in mitosis gene A; ROS, reactive oxygen species; CARD8, caspase recruitment domain-containing protein 8; LPS, lipopolysaccharide; AIM2, absent in melanoma 2; GSDMD, gasdermin D.

Innate immunity is regarded as the first line of defense in the human immune system, responding to PAMPs and DAMPs with the assistance of pattern recognition receptors (PRRs) (Karasawa and Takahashi, 2017). A great number of PRRs are involved in the proinflammatory process *via* directly inducing the formation of the corresponding inflammasome (**Figure 1**).

### 3.1 The NLRP3 inflammasome

#### 3.1.1 The structure of the NLRP3 inflammasome

The NLRP3 inflammasome is an oligomeric intracellular multiprotein complex that contains three parts: NLRP3, apoptosis-associated speck-like protein containing a CARD (ASC), and pro-caspase-1 (Karasawa and Takahashi, 2017). NLRP3 has a general tripartite construction of NOD-like receptors (NLRs) proteins (Kim et al., 2016), comprising the central NOD domain (also known as NAIP, CIITA, HET-E, and TP-2 [NACHT]), the C-terminal leucine-rich repeat (LRR) domain, and the N-terminal pyrin as the effector domain (Ren et al., 2017). The LRR domain usually serves as an adaptor to the NACHT domain and detector of PAMPs and DAMPs in the NLR family (Kim et al., 2016). ASC is a speck-like protein consisting of a C-terminal CARD and an N-terminal PYD (Srinivasula et al., 2002) that performs as the connection between NLRP3 and pro-caspase-1. Pro-caspase-1 can perform auto-cleavage and generate cleaved caspase-1, which is a vital protease in human homeostasis and inflammatory reactions. Caspase-1 forms an active oligomer, cleaving pro-IL-1β and pro-IL-18 into mature IL-1β and IL-18 and promoting their release (Guo et al., 2015).

#### 3.1.2 The process of the NLRP3 inflammasome activation

After TLRs detect PAMPs and DAMPs, the IκB kinase (IKK) complex in the cytoplasm is activated, leading to the phosphorylation, ubiquitination, and degradation of the inhibitor of NF-κB (IκB) and releasing the active NF-κB molecule (Akira et al., 2006; Solt and May, 2008). Then NF-κB enters the nucleus and initiates the transcription of the NLRP3, pro-IL-1β, and pro-IL-18 (Bauernfeind et al., 2009).

After the first stimuli of PAMPs and DAMPs, the NLRP3 just released into the cytoplasm is auto-suppressed and inactive. To generate the NLRP3 inflammasome with full proteolytic activity, the second stimuli are needed (Bauernfeind et al., 2009), mainly consisting of ionic flux, reactive oxygen species (ROS) and mitochondrial dysfunction, and lysosomal damage (Zhou et al., 2011). Under the second stimuli, NLRP3 tends to undergo the conformational change of self-oligomerization. The pyrin domain of NLRP3 binds to ASC, initiating the recruitment of ASC. CARD on ASC connects with pro-caspase-1 *via* the homotypic CARD–CARD interaction, and PYD on ASC also tightly connects with the PYD on the NLRP3 through the similar PYD-PYD interaction (Sborgi et al., 2015; Schmidt et al., 2016; Fernandes-Alnemri et al., 2007). The oligomerization of the pro-caspase-1 on ASC maturates the caspase-1, inducing subsequent inflammatory processes.

#### 3.1.3 The regulation of the NLRP3 inflammasome activation

As mentioned above, the NLRP3 inflammasome activation requires two steps of stimulation. The first signals are mainly PAMPs or DAMPs, activating the PRRs and inducing the transcription of the NLRP3, pro-IL-1β, and pro-IL-18 *via* the NF-κB pathway. The second signals activate NLRP3 and promote the assembly of the inflammasome (Bauernfeind et al., 2009), mainly consisting of ionic flux, reactive oxygen species (ROS) and mitochondrial dysfunction, and lysosomal damage (Zhou et al., 2011).

Ionic flux is the main trigger of the NLRP3 inflammasome activation. The efflux of potassium (K^+^) has been considered a prevalent ion event triggering the activation of the NLRP3 inflammasome. It has been revealed that a low intercellular K^+^ concentration was capable of activating the NLRP3 inflammasome alone, while an increased extracellular K^+^ concentration held up the activation (Mohamed, 2015; Kwak et al., 2018). In addition, ATP is recognized as a vital activator of the NLRP3 inflammasome. However, previous research found that mice with a genetic lack of P2X7 receptor (P2X7R) did not release IL-1β in response to ATP (Solle et al., 2001; Mariathasan et al., 2004). This implies that ATP alone cannot activate the NLRP3 inflammasome. Instead, it relies on P2X7R to mediate the activation. P2X7R is a bi-functional ATP-gated plasma membrane ion channel that allows the efflux of K^+^ and the mobilization of calcium (Ca^2+^) while receiving the stimulus of ATP (Martínez-Cuesta et al., 2020). The mobilization of Ca^2+^ is also a common ionic event in the cell membrane system, participating in multiple intracellular signaling pathways (Clapham, 2007). Inositol 1,4,5-trisphosphate receptor (IP3R) is a Ca^2+^-release channel on the ER. It can be triggered by IP3, which is the downstream product of phospholipase C (PLC)-mediated phosphatidylinositol 4,5-bisphosphate (PIP2) cleavage. Previous evidence showed that inhibiting either IP3R or PLC could lead to the blockade of the NLRP3 inflammasome activation without external stimuli (Lee et al., 2012). Other than K^+^ and Ca^2+^, the ionic flux of sodium (Na^+^) and chloride (Cl^-^) plays a similar function in the NLRP3 inflammasome activation. Na^+^ influx usually leads to water influx and cellular swelling, lowering the intracellular K^+^ concerntration and promoting K^+^ efflux which activates the NLRP3 inflammasome (Schorn et al., 2011). Cl^-^ efflux promotes NLRP3-NEK7 contact and the subsequent NLRP3-ASC complex formation and ASC oligomerization, thus facilitating the NLRP3 inflammasome assembly and activation (Tang et al., 2017).

Mitochondrial dysfunction results in the breakdown of the oxidation respiratory chain reaction, causing oxidative stress to accumulate in terms of mitochondrial ROS (mtROS). ROS is a broad group of substances decomposed by specialized cellular enzymes, including peroxidases and superoxide dismutases (Fidanboylu et al., 2011). It has been proved to be a trigger of the NLRP3 inflammasome activation through experiments with the application of chemical inhibitors (Dostert et al., 2008). Besides, another production of mitochondrial dysfunction is mitochondrial DNA (mtDNA). Multiple screens found that released mtDNA tended to interact with PRRs such as NLRP3 and AIM2. An *in vitro* study with synthesized mtDNA supported the view that released mtDNA in the cytoplasm would be oxidized into oxidized mtDNA, which promoted the NLRP3 inflammasome activation (Zhong et al., 2018).

Lysosomal damage causes two significant consequences: lysosomal acidification and lysosomal content leakage, both of which contribute to the NLRP3 inflammasome activation. Suppressing lysosomal H^+^ flux with a corresponding inhibitor resulted in the inhibition of particulate matter-induced NLRP3 inflammasome activation (Hornung et al., 2008), which indicated that lysosomal acidification was critical to the NLRP3 inflammasome activation. Lysosomal leakage matters are mainly lysosomal enzymes. Cathepsin B is an important lysosomal enzyme that is proven to be essential in the secretion of IL-1β, but not the transcription and maturation. Related findings were obtained from further studies on cathepsins such as cathepsin L, C, S, and X (Orlowski et al., 2015).

### 3.2 The CARD8 inflammasome

The caspase recruitment domain-containing protein 8 (CARD8) inflammasome consists of the sensor CARD8 and the effector pro-caspase-1 (Jin et al., 2022). The sensor CARD8 has two domains: the C-terminal CARD and the N-terminal function-to-find domain (FIIND), which comprises two subdomains named ZU5 and UPA (Taabazuing et al., 2020). Under the inducement of specific PAMPs, the FIIND of CARD8 undergoes degradation in proteasomes and leads to the release of the C-terminal bioactive subunit UPA-CARD, in which the CARD domain recruits pro-caspase-1 through CARD-CARD interaction and then results in the CARD8 inflammasome activation (Taabazuing et al., 2020; Wang and Shan, 2022). Specifically, it has been observed that when CVB3 infected aortic endothelial cells or cardiomyocytes, the CARD8 inflammasome would be activated and accelerate the viral release to neighboring target cells (Wang and Shan, 2022).

### 3.3 The caspase-11 inflammasome

The non-canonical caspase-11 inflammasome robustly mediates anti-bacterial innate immune responses of murine by responding to the bacterial lipopolysaccharide (LPS) (Hagar et al., 2013; Kayagaki et al., 2013). Caspase-11 comprises two domains connected by a CARD domain linker (CDL): an N-terminal CARD and a protease domain, and the protease domain consists of a large subunit and a small subunit separated by an interdomain linker (IDL) (Ross et al., 2022). It has been reported that the CARD of caspase-11 could directly bind to LPS, leading to the oligomerization and activation of the caspase-11 inflammasome (Shi et al., 2014). In another research, caspase-11 gained its basal protease activity by LPS-induced dimerization, which was inadequate for the cleavage of GSDMD (Ross et al., 2018). Following the dimerization, the self-cleavage at site D285 on the IDL of caspase-11 generated the fully active protease species, which could cleave GSDMD and promote pyroptosis (Ross et al., 2018). Compared to canonical inflammasomes such as the NLRP3 inflammasome, the activation process of the caspase-11 inflammasome shows distinct features: the caspase-11 directly binds to the LPS and assembles without the aid of signaling sensors or adaptors (Shi et al., 2014; Ross et al., 2018). Furthermore, caspase-11 exists in murine, while caspase-4 and caspase-5 are the human orthologs of murine caspase-11 (Lamkanfi et al., 2002). It has been observed that caspase-4 and caspase-5 could also bind to LPS and be activated by the LPS inducement (Shi et al., 2014), which indicated that caspase-4 and caspase-5 might undergo their activation in similar ways as caspase-11.

### 3.4 The AIM2 inflammasome

Absent in melanoma 2 (AIM2), a double-stranded DNA sensor, comprises a PYD and a C-terminal HIN domain, which can recognize and combine with autologous or foreign DNA (Hornung et al., 2009). When the HIN domain combines with DNA, the PYD interacts with the PYD of the adaptor ASC, and the CARD of ASC links to the CARD of pro-caspase-1, promoting the assembly and activation of the AIM2 inflammasome (Bürckstümmer et al., 2009). The oligomerization of caspase-1 in the AIM2 inflammasome improves its ability to process pro-IL-1β into bioactive IL-1β. Besides, caspase-1 can also split gasdermin D (GSDMD) into two fragments, and the N-terminal fragment forms large pores on the plasma membrane, leading to IL-1β release and cell pyroptosis (Lugrin and Martinon, 2018).

## 4 The regulation of the inflammasome activation and inhibition in VMC

During the infectious phase of VMC, multiple categories of molecules of either host cells or viruses participate in the regulation of the inflammasome activation and inhibition, thus influencing the development of VMC.

### 4.1 The NLRP3 inflammasome

Extensive research has demonstrated that the NLRP3 inflammasome is the main inflammasome involved in CVB3-induced VMC (Wang et al., 2018; Wang et al., 2014; Tschöpe et al., 2017; Wang et al., 2019; Tong et al., 2020; Liu et al., 2022). The NLRP3 inflammasome is activated through a variety of pathways during CVB3 infection, promoting the production and secretion of IL-1β and IL-18 and exacerbating pyroptosis. Several mechanisms of the regulation of the NLRP3 inflammasome activation in CVB3-induced VMC are discussed below.

#### 4.1.1 PRRs

PRRs including nucleotide-binding oligomerization domain 2 (NOD2) and TLR4 may play significant roles in the NLRP3 inflammasome activation in CVB3-induced VMC. It has been reported that in the context of CVB3-induced VMC, NOD2 knockout mice manifested lower NLRP3 and ASC levels in the left ventricular and serum IL-1β levels in comparison with wild-type mice. Furthermore, the NOD2-mediated NLRP3 inflammasome activation has been demonstrated to be CVB3-dependent (Tschöpe et al., 2017). Besides, in mice with CVB3-induced VMC, TLR4 deficiency has been demonstrated to alleviate the production of two typical inflammasome related cytokines, IL-1β and IL-18 (Fairweather et al., 2003). Extensive colocalization between TLR4 and enterovirus capsid protein VP1 has been detected in the cytoplasm of cardiomyocytes obtained from DCM patients (Satoh et al., 2004), which indicated the possible interaction between the virus and TLR4 in cardiomyocytes. However, the detailed mechanisms of NOD2/TLR4-related NLRP3 inflammasome activation need further elucidation.

#### 4.1.2 K^+^ efflux

K^+^ efflux robustly promotes the NLRP3 inflammasome activation in CVB3-induced VMC. It has been demonstrated that in CVB3-infected cardiomyocytes, the NLRP3 inflammasome activation accounted for CVB3-induced IL-1β secretion, and once K^+^ efflux was inhibited by culturing cardiomyocytes in K^+^-rich medium, the CVB3-induced caspase-1 activity and IL-1β secretion were significantly down-regulated (Wang et al., 2014). Furthermore, to avoid the possibility that the functions of cardiomyocytes might be influenced not only by the K^+^ channels but also some voltage-gated Na^+^ channels, glibenclamide, an ATP-sensitive K^+^-channel inhibitor, was used to block K^+^ efflux and, in turn, robustly suppressed IL-1β secretion of CVB3-infected cardiomyocytes (Wang et al., 2014). These data demonstrated the role of K^+^ efflux in the NLRP3 inflammasome activation in CVB3-induced VMC.

#### 4.1.3 Calpain-1

The calpains are a conserved family of Ca^2+^-dependent cysteine proteases expressed generally in all cells (Goll et al., 2003), and calpain-1 has been demonstrated to be activated in CVB3-induced VMC to activate the NLRP3 inflammasome through promoting mitochondrial dysfunction and mtROS production (Liu et al., 2022). Liu et al. have found that in the cardiomyocytes of mice with CVB3-induced VMC, calpain was detected to be activated. The NLRP3 inflammasome activation triggered by CVB3 infection could be inhibited by overexpression of calpastatin, a natural and specific endogenous inhibitor of calpain activity. Meanwhile, the NLRP3 inflammasome activation could also be inhibited by mito−TEMPO, a mitochondrial-targeted antioxidant in cardiovascular conditions that could reduce CVB3-induced mtROS level (Liu et al., 2022). The relationship between calpain-1 and mtROS in CVB3-induced VMC could be further elucidated by the fact that mitochondrial function was improved when calpain activity was inhibited by calpastatin *in vivo*, and that the GSDMD N-terminus and caspase-1 exacerbated by CVB3-induced calpain-1 overexpression could be suppressed by mito-TEMPO (Liu et al., 2022). As the results above showed, excessive calpain-1 activity could increase mtROS, which activated the NLRP3 inflammasome in mice with CVB3-induced VMC (Liu et al., 2022). Furthermore, under CVB3 stimulation, calpain-1 was observed accumulating in the mitochondria and reducing the expression of ATP synthase-α (ATP5A1) (Liu et al., 2022), which was significant for mitochondrial function (Ni et al., 2016), and the reduction of ATP5A1 led to the NLRP3 inflammasome activation in cardiomyocytes (Liu et al., 2022). In summary, in the context of CVB3-induced VMC, CVB3-induced calpain-1 translocation from the cytoplasm to mitochondria reduces the expression of ATP5A1, a subunit of mitochondrial ATP synthase 1. The decrease of ATP5A1 impairs mitochondrial function and increases mtROS production, which subsequently activates the NLRP3 inflammasome and induces the pyroptosis of cardiomyocytes.

#### 4.1.4 Cathepsin B

Cathepsins are a family of lysosomal cysteine proteases participating in multiple cellular processes, among which cathepsin B may play an important role in CVB3-induced VMC. Wang et al. have observed that cathepsin B was activated in both acute and chronic stages of CVB3-induced VMC in mice and intensified cardiomyocyte damage (Wang et al., 2018). Furthermore, they found that the activated cathepsin B elevated protein levels of NLRP3, ASC, caspase-1 p20, and IL-1β. More importantly, the activated cathepsin B exacerbated caspase-1 activity and myocardial pyroptosis, which were both significant outcomes of the NLRP3 inflammasome activation (Wang et al., 2018). The results above demonstrated that activated cathepsin B promoted the NLRP3 inflammasome activation and exacerbated myocardial symptoms in CVB3-induced VMC.

#### 4.1.5 MicroRNA

In recent years, microRNAs (miRs) have been identified to regulate gene expression at the transcriptional and post-transcriptional levels (Li et al., 2019). MiRs induce mRNA degradation or terminate transcription by binding to the 3’-untranslated region (UTR) of mRNAs (Bartel, 2004). Multiple miRs have been associated with VMC, including miR-1, miR-15, miR-21, miR-146, miR-155, miR-221, miR-222, and miR-223 (Chau et al., 2007; Rose, 2009; Blauwet and Cooper, 2010; Smith et al., 2017; Tong et al., 2020; Xue et al., 2020). Among them, miR-15 and miR-223 play important roles in regulating the NLRP3 inflammasome activation in CVB3-induced VMC.

An experiment demonstrated that the miR-15-NLRX1 axis was involved in the regulation of the NLRP3 inflammasome activation in CVB3-induced VMC (Tong et al., 2020). Nucleotide-binding domain and leucine-rich-repeat-containing family member X1(NLRX1), a member of the NOD-like receptor family, has been proved closely associated with inflammatory diseases (Karin et al., 2006; Kang et al., 2015). The expression of miR-15 dramatically increased after CVB3 infection, which down-regulated the expression of NLRX1. Once the expression of the NLRX1 protein was restrained by increased miR-15, the production of IL-1β and IL-18 increased through the NLRP3 inflammasome activation pathway, followed by intensified inflammatory responses, reduced cell viability, and promoted cell apoptosis. As a result, myocardial injury was aggravated. In summary, during the CVB3-induced VMC, the increased miR-15 represses NLRX1, contributing to the NLRP3 inflammasome activation. This implies a novel pathway for the NLRP3 inflammasome activation.

In addition to miR-15, the role of miR-223 is also remarkable. In CVB3-induced VMC mice, the researchers found that the levels of long non-coding RNA (lncRNA) maternally expressed gene 3 (MEG3) in the myocardium increased, resulting in miR-223 down-regulation. MiR-223 targets the mRNA of the TNF receptor‐associated factor 6 (TRAF6), a ubiquitin E3 ligase essential for IKK activation in the IL-1 and TLR pathways (Lomaga et al., 1999; Naito et al., 1999; Xue et al., 2020). Furthermore, the researchers found that the inhibition of miR-223 up-regulated TRAF6 (Xue et al., 2020). The up-regulation of TRAF6 activated the NF‐κB pathway to promote the NLRP3 inflammasome activation, elevating the protein levels of related inflammatory cytokines (Xue et al., 2020). Besides, another research demonstrated that in CVB3-infected mice, the treatment of A20, a TRAF6 inhibitor also known as tumor necrosis factor alpha induced protein 3 (TNFAIP3), effectively alleviated CVB3-induced VMC by down-regulating TRAF6 (Gui et al., 2012). The role of ubiquitination in the pathogenic process of VMC is further established, which is consistent with previous findings that ubiquitination plays significant roles in regulating the inflammasome function (Xu and Núñez, 2022).

As discussed above, multiple molecules participate in the regulation of the NLRP3 inflammasome activation in the VMC, including PRRs, K^+^, calpain-1, cathepsin B and microRNA, all of which participate in the development of the VMC. Interestingly, it has been reported that CVB3 protease 3C could degrade NLRP3 (Wang et al., 2019). Meanwhile, CVB3 protease 3C could also degrade receptor-interacting protein (RIP)1/RIP3, two molecules contributing to the NLRP3 inflammasome activation through the RIP1-RIP3-dynamin-related protein 1(DRP1)-NLRP3 inflammasome pathway (Wang et al., 2014; Wang et al., 2019). CVB3 might adopt this cleavage method to ameliorate the NLRP3 inflammasome activity in order to escape the host immune response. However, it is more widely observed that, upon CVB3 infection, various kinds of molecular events promote rather than inhibit the activation of the NLRP3 inflammasome, thereby exacerbating the symptoms of VMC.

### 4.2 The CARD8 inflammasome

Apart from the NLRP3 inflammasome, the CARD8 inflammasome can be activated by CVB3 proteases and participate in the progress of CVB3-induced VMC. Nadkarni et al. have demonstrated that in CVB3-infected human aortic endothelial cells (HAECs), CVB3 2A protease and 3C protease could cleave the CARD8 N-terminus. The cleaved CARD8 neo-N-terminus underwent degradation by proteasomes, releasing the bioactive UPA-CARD domain, which could interact with pro-caspase-1 and promote the CARD8 inflammasome assembly. Then the CARD8 inflammasome facilitated GSDMD cleavage, cell pyroptosis, and viral release to adjacent target cells (Nadkarni et al., 2022). Importantly, the researchers also demonstrated that CARD8 knockout HAECs protected the underlying cardiomyocytes from CVB3 infection (Nadkarni et al., 2022), indicating that the CARD8 inflammasome activity in CVB3-infected endothelial cells might increase the risk of viral infection in myocardium and even cause VMC. Furthermore, in CVB3-infected cardiomyocytes, knockout of CARD8 led to a reduction of cleaved GSDMD and ameliorated cell death (Nadkarni et al., 2022), suggesting that the CARD8 inflammasome also directly participated in the pyroptotic progress of CVB3-infected cardiomyocytes. In summary, the CARD8 inflammasome may participate in pyroptosis in different cell types and exacerbate the VMC after the viral infection.

### 4.3 The caspase-11 inflammasome

Besides the canonical inflammasomes mentioned above, the non-canonical caspase-11 inflammasome also participates in the VMC. Calpain has been frequently observed up-regulated in hearts of mice with CVB3-induced VMC (Yu et al., 2020; Liu et al., 2022). Yu et al. have found that calpain was strongly activated and exacerbated pyroptosis in hearts of CVB3-infected mice (Yu et al., 2020), while in the calpastatin transgenic mouse strain (Tg-CAST) that overexpressed calpastatin, a natural and specific endogenous inhibitor of calpain, the severity of VMC was significantly lower than in the wild-type mice (Yu et al., 2020). The researchers further found that in the hearts of CVB3-infected mice, the inhibition of calpain not only down-regulated the expression of canonical NLRP3 inflammasome components, including NLRP3, ASC, and caspase-1, but also significantly suppressed the expression of caspase-11 (Yu et al., 2020). The results suggested that the activation of the non-canonical caspase-11 inflammasome might participate in the VMC as a downstream molecular event of calpain activation. However, more evidence is needed to elucidate the detailed mechanisms of caspase-11 inflammasome activation in the context of VMC. Although the involvement of the caspase-4 inflammasome in myocardial diseases such as myocardial reperfusion-induced microvascular injury has been reported (Sun et al., 2021), the roles of the caspase-4 inflammasome and the caspase-5 inflammasome in VMC have not been established and need more exploration.

### 4.4 The AIM2 inflammasome

The AIM2 inflammasome activation has been reported to exacerbate symptoms in multiple cardiac conditions, such as heart failure and myocardial infarction (Onódi et al., 2021; Liao et al., 2022). Although there has been no evidence that the AIM2 inflammasome directly participated in the VMC, Furrer et al. reported that AIM2 could inhibit NF-κB p65 acetylation and phosphorylation in cardiomyocytes, thus directly suppressing proinflammatory cytokine transcription (Furrer et al., 2016). The researchers also found that AIM2 inhibited inflammatory cytokine (including IL-6, IP-10, and TNF-α) transcription in cardiomyocytes indirectly *via* limiting phosphorylation of signal transducer and activator of transcription 1 (STAT1), an important regulator in the NF-κB pathway (Furrer et al., 2016). Interestingly, in cardiomyocytes, knockout of caspase-1 and AIM2 manifested the same profile of proinflammatory cytokine transcription as the mere knockout of AIM2 (Furrer et al., 2016), indicating that the AIM2-mediated suppression on proinflammatory cytokine production in cardiomyocytes possibly followed a caspase-1/inflammasome-independent manner (Furrer et al., 2016). The research above suggested that, apart from acting as an inflammasome sensor, AIM2 might play other potential roles in VMC. Besides, AIM-2 co-immunization has been reported to ameliorate symptoms of CVB3-induced VMC *via* multiple mechanisms, such as promoting specific multifunctional CD8 T cell induction, facilitating protective secretory immunoglobulin A (SIgA) response and increasing prophylactic efficacy of chitosan-DNA vaccine (Chai et al., 2014; Chai et al., 2015), all of which seemingly showed the potential of AIM2 in preventing VMC. However, the mechanisms underlying the AIM2 activity in VMC are not fully established, and further investigations are needed.

## 5 The effects of the inflammasome activation in VMC

**Figure 2:**
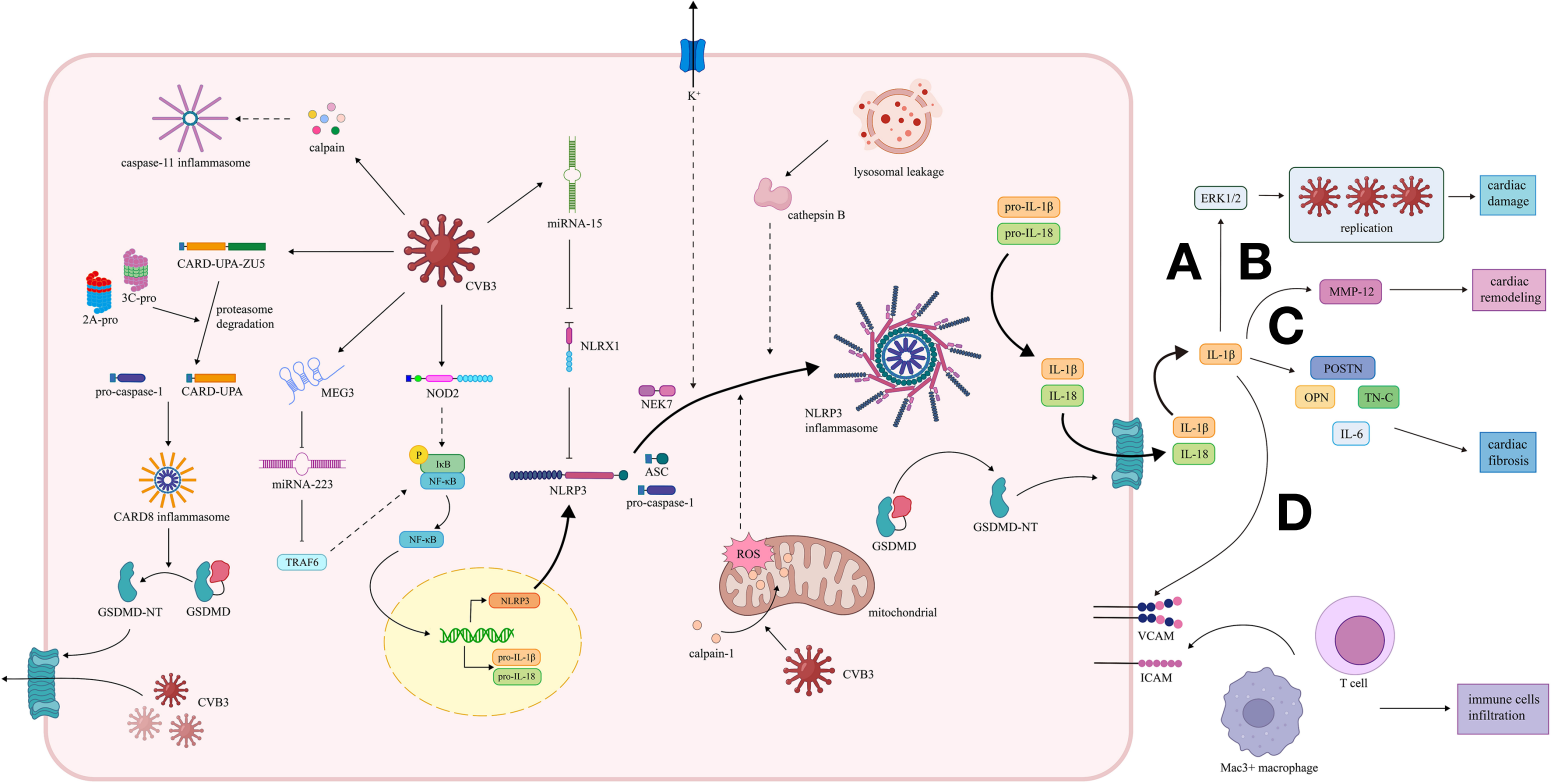
Activation and effect of inflammasome pathway in VMC induced by CVB3. (1) CARD8 inflammasome pathway. The 2A and 3C proteases of CVB3 promote the cleavage of CARD8 to form the CARD8 inflammasome, which provides a platform for the maturation of pro-caspase-1. Caspase-1 cleaves GSDMD, and the transfer of the GSDMD N-terminal fragment to the cell membrane increases the permeability of the cell membrane, thereby promoting the release of replication-generated CVB3. (2) NLRP3 inflammasome pathway. CVB3 is recognized by NOD2, activating the NF-κB pathway, leading to the transcription of NLRP3, pro-IL-1 and pro-IL-18. CVB3-induced NLRP3 inflammasome activation in VMC is mediated by potassium efflux, release of cathepsin B caused by lysosomal damage, and ROS and mitochondrial dysfunction due to the transfer of calpain-1 to mitochondria. The NLRP3 inflammasome promotes the maturation of pro-IL-1β and pro-IL-18 and the cleavage of GSDMD. IL-1β plays a significant role in the development of VMC. MicroRNAs, including miR-15/223, are also involved. MiR-15 is up-regulated to repress NLRX1, while miR-223 is down-regulated to elevate the TRAF6, both contributing to the NLRP3 inflammasome activation. **(A)** IL-1β induces high expression of ERK1/2, facilitating viral replication, leading to damage and inflammation of cardiac tissue. **(B)** IL-1β promotes transcription of MMP12 in myocytes, thus leading to cardiac remodeling. **(C)** IL-1β promotes the expression of OPN, TN-C, POSTN, and IL-6, contributing to cardiac fibrosis. **(D)** IL-1β enhances the expression of ICAM and VCAM, promoting the infiltration of immune cells, including Mac3+ macrophages and CD3+ T cells. (3) Caspase-11 inflammasome pathway. The calpain activation induced by CVB3 can also activates caspase-11 inflammasome, which may participate in the pathogenesis of VMC. VMC, viral myocarditis; CVB3, coxsackie virus B3; CARD8, caspase recruitment domain-containing protein 8; GSDMD, gasdermin D; NLRP3, nucleotide oligomerization domain-, leucine-rich repeat-, and pyrin domain-containing protein 3; NOD2, nucleotide-binding oligomerization domain 2; TLR, toll-like receptors; NF-κB, nuclear factor κ-light-chain enhancer of activated B cells; ROS, reactive oxygen species; miR, microRNA; NLRX1, Nucleotide-binding domain and leucine-rich-repeat-containing family member X1; TRAF6, TNF receptor‐associated factor 6; ERK 1/2, extracellular signal-regulated kinases 1 and 2; MMP12, matrix metalloproteinase 12; OPN, osteopontin; TN-C, tenascin C; POSTN, periostin; ICAM, intercellular cell adhesion molecule; VCAM, vascular cell adhesion molecule.

Among the downstream molecules of inflammasomes, IL-1β participates in multiple cellular events in both infectious and post-infectious phases, indicating that inflammasomes can influence the development of VMC through the secretion of IL-1β. (**Figure 2**).

### 5.1 The effects of IL-1β secretion in the infectious phase of VMC

The activation of extracellular signal-regulated kinases 1 and 2 (ERK1/2), two significant molecules in IL-1β pathway, has been demonstrated to facilitate viral replication, exacerbate viral infectivity, and decrease cell survival in Hela cells (Luo et al., 2002; Weber et al., 2010). High levels of ERK1/2 were observed in the hearts of CVB3-infected mice, and ERK1/2 expression was detected spatially identical with myocardial damage and inflammation (Kraft et al., 2019), which were consistent with the results in Hela cells. However, in mice with CVB3-induced VMC, neutralization of IL-1β with IL-1β antibody led to the reduction of both viral replication and myocardial damage (Kraft et al., 2019). More importantly, IL-1β blockade robustly down-regulated the ERK1/2 expression (Kraft et al., 2019), which provided a reasonable explanation for the ameliorated viral replication. It can be deduced from the facts above that IL-1β matured by inflammasomes may exacerbate symptoms of CVB3-induced VMC by promoting viral replication.

### 5.2 The effects of IL-1β secretion in the post-infectious phase of VMC

#### 5.2.1 Immune cell infiltration

The intercellular cell adhesion molecule (ICAM) and vascular cell adhesion molecule (VCAM) are vital for the adhesion and infiltration of leukocytes (Seko et al., 1996). It has been reported that both ICAM-1 and VCAM-1 were up-regulated in cardiomyocytes and participated in the development of myocarditis in mice with CVB3-induced VMC (Seko et al., 1993; Seko et al., 1996). Kraft et al. found that in the hearts of CVB3-infected mice, IL-1β antibody treatment in the acute stage of VMC down-regulated adhesion molecules, including ICAM-1 and VCAM-1, in the chronic stage of VMC, and inhibited infiltration of immune cells, including Mac3+ macrophages and CD3+ T cells, thus alleviating the CVB3-induced inflammation (Kraft et al., 2019). It is reasonable to deduce that in CVB3-induced VMC, IL-1β secretion *via* the inflammasome pathway can exacerbate the inflammatory responses by promoting the adhesion molecule expression and immune cell infiltration, which might promote enduring inflammation and cardiac symptoms.

#### 5.2.2 Fibrosis

In hearts, IL-6 is mainly produced by macrophages and fibroblasts, and it can be up-regulated under IL-1β stimulation (Ma et al., 2012; Althof et al., 2018; Kishimoto and Kang, 2022). The reduction of IL-6 has been reported to ameliorate cardiac fibrosis (Ma et al., 2012; Segers et al., 2018). Kraft et al. reported that in mice with CVB3-induced VMC, neutralization of IL-1β by IL-1β antibody in the acute stage of VMC robustly suppressed both the IL-6 expression and collagen type I deposition and inhibited the cardiac fibrosis process in the chronic stage (Kraft et al., 2019), which was consistent with previous publications. These studies indicate that the inflammasome-induced IL-1β secretion may promote IL-6 expression, thus exacerbating cardiac fibrosis during the chronic VMC.

Matricellular proteins are nonstructural proteins binding to the extracellular matrix and participate in the regulation of cell survival, differentiation, and mobilization (Zohar et al., 2004; Harris et al., 2011). Among them, osteopontin (OPN), tenascin C (TN-C), and periostin (POSTN) can accelerate cardiac fibrosis (López-Sánchez et al., 2017; Abdelaziz Mohamed et al., 2019; Mohammadzadeh et al., 2020). It has been demonstrated that the three matricellular proteins mentioned above were highly expressed in the hearts of CVB3-infected mice, while they were all down-regulated in the chronic stage by treating with IL-1β antibody during the acute stage of VMC (Kraft et al., 2019). Therefore, the inflammasome-induced IL-1β secretion may exacerbate cardiac fibrosis by up-regulating the matricellular proteins.

#### 5.2.3 Cardiac remodeling

Matrix metalloproteinases (MMPs) are a group of proteolytic enzymes whose main function is the regulation of extracellular matrix reconstruction (Spinale, 2007). Previous studies showed vital involvement of MMPs in the cardiac remodeling process after the CVB3 infection (Westermann et al., 2010). Among multiple MMPs, MMP12 was observed remaining at a high transcription level while the other MMPs decreased after the acute stage of CVB3-induced VMC (Westermann et al., 2010), which indicated the possible involvement of MMP12 in the cardiac remodeling process. Kraft et al. found high expression levels of MMP12 in the hearts of mice during the chronic stage of CVB3-induced VMC, while MMP12 expression in the chronic stage was down-regulated significantly by IL-1β antibody in the acute stage (Kraft et al., 2019). The results above indicated that IL-1β secretion *via* the NLRP3 inflammasome pathway might contribute to the cardiac remodeling process by regulating the MMP12.

## 6 Roles of inflammasomes in COVID-19-related myocarditis

A wide variety of cardiovascular diseases after SARS-Cov-2 infection have been reported, including myocarditis (Boukhris et al., 2020; Irabien-Ortiz et al., 2020; Sawalha et al., 2021), and the NLRP3 inflammasome may be activated by SARS-Cov-2 infection and participate in the progression of Corona Virus Disease 2019 (COVID-19)-related myocarditis. Viral presence has been observed in the myocardium after SARS-Cov-2 infection by endomyocardial biopsy (EMB) and autopsy (Albert et al., 2020; Fox et al., 2020; Lindner et al., 2020), which indicated possible viral infection of cardiomyocytes in the COVID-19 context. Angiotensin-converting enzyme 2 (ACE2) is a host cellular receptor that can bind to the spike (S) protein of SARS-Cov-2 and facilitate viral entry into the host cell with the aid of TMPRSS2 (Fox et al., 2020; Quagliariello et al., 2020). Both ACE2 and TMPRSS2 have been demonstrated to be expressed in the heart (Feng et al., 2021; Chen et al., 2020; Clerkin et al., 2020; Nicin et al., 2020). Meanwhile, ACE2 can also cleave angiotensin (Ang) II to generate Ang- (1-7), thus reducing the level of Ang II (Santos et al., 2018). From the facts above, it is reasonable to deduce that with increasing viral infection and replication in myocardium, the S protein-ACE2 interaction and internalization of ACE2 after the interaction are enhanced, thus ameliorating the ACE2-Ang II interaction. The possible inhibition of the ACE2-Ang II interaction may suppress Ang II degradation and lead to Ang II accumulation. Importantly, it has been demonstrated that excessive Ang II could induce cardiomyocyte pyroptosis and cardiac fibrosis through activating the NLRP3 inflammasome (Pinar et al., 2020; Zhu et al., 2021), which manifested similar features of myocarditis. Thus, it is reasonable to suggest that SARS-Cov-2 infection may participate in COVID-19-related myocarditis by activating the NLRP3 inflammasome.

Cytokine release syndrome is a systemic inflammatory response triggered by adverse external factors such as viral infection (Shimabukuro-Vornhagen et al., 2018). The migration of activated CD8 T cells to the heart triggers cytokine release syndrome, leading to cardiac injury. This inflammatory cytokine storm is considered to be the main mechanism of acute COVID-19-related myocarditis (Chimenti et al., 2022). Furthermore, possible roles of the NLRP3 inflammasome in this process were also well-discussed. After SARS-CoV-2 infection, the NLRP3 inflammasome can be activated by multiple stimuli, including Ca^2+^ mobilization, K^+^ efflux, ROS production, Ang II accumulation induced by the S protein-ACE2 pathway, and the complement cascade induced by SARS-CoV-2 infection (Zhao et al., 2021). As the NLRP3 inflammasome-induced proinflammatory cascade progresses, IL-1β and IL-18 promote the release of other cytokines, including IL-6, which is hypothesized to be a central mediator of cytokine storm. IL-6 coordinates the proinflammatory responses of immune cells to further promote the release of inflammatory cytokines and achieve the positive feedback regulation that induces the cytokine storm, thus contributing to the cytokine release syndrome (Coomes and Haghbayan, 2020). Moreover, it has been suggested that suppressing the NLRP3 inflammasome with inhibitors such as MCC950 could effectively prevent the development of the cytokine storm. This study demonstrated the significant participation of the NLRP3 inflammasome in inducing the inflammatory cytokine storm, the main pathogenic mechanism of COVID-19-related myocarditis (Ratajczak et al., 2021), and provided ideas for the treatment of COVID-19-related myocarditis.

## 7 Potential therapies targeting inflammasomes and related pathways

The well-established roles of inflammasomes, especially the NLRP3 inflammasome, in the VMC provide multiple ideas for possible therapies for VMC targeting the inflammasome pathway. Several drugs inhibiting the NLRP3 inflammasome or its downstream IL-1β are reported to be promising therapies for VMC in a variety of case reports, clinical trials, and animal experiments (**Table 1**).

**Table 1:** Overview of case reports, clinical trials and animal experiments targeting the inflammasome pathway to treat VMC.

Case report												
Case reports	Intervention	Target of therapy	Age	Nationality	Diagonosis	Medical history	Management	Outcome	Adverse outcome	Year of publication	PMID	
Treating Life-Threatening Myocarditis by Blocking Interleukin-1	anakinra	Inhibiting IL-1β	36 years	Italy	fulminant myocarditis secondary to H1N1 influenza virus infection	none	100mg/d; day 6-day 10	excellent health at 12 months ; complete resolution	none	2016	27031379
Case Report: Life-Threatening Macrophage Activation Syndrome With Fulminant Myocarditis Successfully Rescued by High Dose Intravenous Anakinra	canakinumab	Inhibiting IL-1β	2 years	Italy	acute myocarditis; systemic onset Juvenile Idiopathic Arthritis	none	4mg / Kg q4wk-4mg / Kg q6wk; 24 months from 6 months after discharge	clinical remission	unknown	2021	33537271
Paediatric viral myocarditis successfully treated with IFN beta-1b and corticoids	IFN-β-1b	Inhibiting NLRP3 inflammasome activation	6 months	Spain	acute myocarditis with PVB-19	unknown	20 µg/kg/day on Monday, Wednesday and Friday ; six months from the day 3	myocarditis resolved after months	none	2020	33403664
Paediatric viral myocarditis successfully treated with IFN beta-1b and corticoids	IFN-β-1b	Inhibiting NLRP3 inflammasome activation	9 months	Spain	acute myocarditis with PVB-19	diarrhoea	20 µg/kg/day on Monday, Wednesday and Friday ; six months from the day 3	myocarditis progressively improved	flu-like syndrome	2020	33403664
Successful Treatment of Enterovirusinduced Myocarditis With Interferon-α	IFN-α-2a	Inhibiting NLRP3 inflammasome activation	65 years	Italy	active myocarditis with enterovirus	Churg-Strauss disease with bronchial asthma, eosinophilia. Etc	6 million IU every other day for 12 months	stable clinically and hemodynamically	Asthenia and nausea	2003	12581773
Successful Treatment of Enterovirusinduced Myocarditis With Interferon-α	IFN-α-2a	Inhibiting NLRP3 inflammasome activation	37 years	Italy	active lymphocytic myocarditis with CVA/CVB	none	4.5 million IU every other day six months from the month 3	asymptomatic	mild nausea and thrombocytopenia	2003	12581773
Clinical trial												
Clinical trials	Intervention	Target of therapy	Number of patients	Major outcomes	Major serious adverse outcomes	Results first posted year	PMID or NCT					
Anakinra Versus Placebo for the Treatment of Acute MyocarditIS (ARAMIS)	anakinra	Inhibiting IL-1β	120	Number of days alive free of any myocarditis complications	unknown	unpublished	NCT03018834				
Canakinumab in Covid-19 Cardiac Injury (The Three C Study)	canakinumab	Inhibiting IL-1β	45	Number of Participants With Clinical Improvement at Day 14	unknown	2021	NCT04365153				
IFN and thymic hormones in the therapy of human myocarditis and idiopathic dilated cardiomyopathy	IFN-α	Inhibiting NLRP3 inflammasome activation	40	unknown	none	1995	8682086				
Interferon-beta treatment eliminates cardiotropic viruses and improves left ventricular function in patients with myocardial persistence of viral genomes and left ventricular dysfunction	IFN-β	Inhibiting NLRP3 inflammasome activation	22	unknown	none	2003	12771005				
Animal experiments												
Animal experiments	Intervention	Target of therapy	Year of publication	PMID								
Blocking the IL-1β signalling pathway prevents chronic viral myocarditis and cardiac remodeling	Canakinumab	Inhibiting IL-1β	2019	30673858							
Mitochondrial calpain-1 activates NLRP3 inflammasome by cleaving ATP5A1 and inducing mitochondrial ROS in CVB3-induced myocarditis	MCC950	Inhibiting NLRP3 inflammasome activation	2022	35997820							
Microtubule-driven spatial arrangement of mitochondria promotes activation of the NLRP3 inflammasome	Colchicine	Inhibiting NLRP3 inflammasome activation	2013	23502856							
Colchicine prevents disease progression in viral myocarditis via modulating the NLRP3 inflammasome in the cardiosplenic axis	Colchicine	Inhibiting NLRP3 inflammasome activation	2022	35178861							
Colchicine aggravates coxsackievirus B3 infection in mice	Colchicine	Inhibiting NLRP3 inflammasome activation	2016	27140338							
IL-37 alleviates Coxsackievirus B3-induced viral myocarditis via inhibiting NLRP3 inflammasome-mediated pyroptosis	Interleukin-37	Inhibiting NLRP3 inflammasome activation	2022	36418383							
Type I IFN inhibits interleukin-1 production and inflammasome activation	IFN-β, IFN-α, IFN-γ	Inhibiting NLRP3 inflammasome activation	2011	21349431							
IFN-γ Protects against Chronic Viral Myocarditis by Reducing Mast Cell Degranulation, Fibrosis, and the Profibrotic Cytokines Transforming Growth Factor-β1, Interleukin-1β, and Interleukin-4 in the Heart	IFN-γ-deficient	Inhibiting NLRP3 inflammasome activation	2004	15579433							

### 7.1 Anakinra

Anakinra is the recombinant form of the naturally occurring IL-1 receptor antagonist (IL-1Ra) and inhibits the activity of both IL-1α and IL-1β. The effectiveness of anakinra in the treatment of myocarditis has been revealed by many animal experiments and case reports, indicating the possible involvement of the NLRP3 inflammasome/IL-1β pathway. As far as we know, the earliest case report of anakinra applied to myocarditis dates back to 2016. A previously healthy 36-year-old Italian woman was admitted to the hospital due to fulminant myocarditis. Because previous treatment was ineffective, from the sixth day of admission, she was given anakinra 100 mg daily, and a dramatic remission was observed within 24 hours. The fever was alleviated and the neutrophil count reduced, along with the normalization of CRP, troponin T, ECG, and LVEF. With 4 days of anakinra treatment, sustained clinical improvement allowed weaning from ECMO and removal of the percutaneous LVAD (Cavalli et al., 2016). Yoshihiro Noji et al. accordingly suggested that anakinra targeted IL-1 to treat fulminant myocarditis (Noji, 2016).

The anakinra vs. Placebo for the Treatment of Acute Myocarditis (ARAMIS) trial (ClinicalTrials.gov identifier: NCT03018834) has been completed as of June 15, 2022. Although the results have not been posted yet, the clinical application of anakinra in acute myocarditis is promising.

In addition, the safety of anakinra has been demonstrated by extensive research. It is significant to know that anakinra is associated with an increased risk of infection (mainly upper respiratory infections), but not with increased infection-related mortality (Fleischmann et al., 2006). The evidence above indicates that anakinra may have great potential in the treatment of VMC.

### 7.2 Canakinumab

Canakinumab is an IL-1β-neutralizing antibody that reduces inflammation in patients with autoimmune diseases. Compared with anakinra, it has a longer half-life and can be administered once a month, showing better clinical operability (Abbate et al., 2020). In the treatment of heart diseases, canakinumab is effective in reducing myocardial infarction in high-risk groups (Ridker et al., 2017).

In a CVB3-induced VMC mouse model, canakinumab’s counterpart was shown to prevent chronic VMC and cardiac remodeling. A total of 36 ABY/SnJ mice were divided into three control groups and three experimental groups. The control groups were infected with CVB3 alone, while the experimental groups were infected with CVB3 first, followed by neutralizing IL-1β with antibodies on days 1–14, 3–14, and 14–28, respectively. Significant reductions in myocardial injury and inflammation were observed in all three experimental groups. At 28 days post-infection, robust cardiac fibrosis and remodeling were observed in the three control groups, whereas the three corresponding experimental groups all showed a marked reduction in the degree of fibrosis during the same period (Kraft et al., 2019).

In a case report of fulminant myocarditis, significant remission was observed when anakinra was applied. After discharge, anakinra was switched to canakinumab because of the difficulty in maintaining daily injections of anakinra. Canakinumab was initiated six months after discharge at a dose of 4 mg/Kg q4wk and then gradually tapered down. Up to 24 months, the disease had been in clinical remission on medication (canakinumab 4 mg/Kg q6wk) (Meneghel et al., 2020). This case not only revealed canakinumab’s effectiveness, but also highlighted its advantage of less frequent injections than anakinra, which had a shorter half-life. In addition, a clinical trial, Canakinumab in COVID-19 Cardiac Injury (The Three C Study), was conducted in 2021 and revealed the role of canakinumab in the treatment of myocardial injury in the context of COVID-19 (ClinicalTrials.gov Identifier: NCT04365153). It indicated that canakinumab could ameliorate SARS-CoV-2-associated acute myocardial injury, but the doses remain to be explored and the side effects are less clear. In summary, it is suggested that canakinumab has great potential in the treatment of VMC. Nevertheless, more clinical studies are needed to explore the efficacy and safety of canakinumab in treating VMC, in order to better guide its application in the management of VMC patients.

### 7.3 MCC950

MCC950, also known as CRID3 and CP-456,773, is a widely studied specific NLRP3 inflammasome inhibitor. Liu et al. proved that in mice with CVB3-induced VMC, intra-peritoneal injection of MCC950 could effectively inhibit CVB3-induced pyroptosis and inflammation by inhibiting the NLRP3 inflammasome activation pathway. Six 4 or 5-week-old male mice were equally divided into three groups: the sham group injected with PBS; the CVB3 group injected with CVB3; and the MCC950+CVB3 group injected with 10 mg/kg MCC950 daily extraperitoneal for six consecutive days and infected with CVB3 on the second day from MCC950 injection initiation. Western blot analysis indicated that the MCC950+CVB3 group showed a significant reduction in GSDMD N-terminus and cleaved caspase-1 compared with the CVB3 group. Moreover, cardiomyocyte viability was higher in the CVB3 + MCC950 group than in the CVB3 group (Liu et al., 2022). This revealed the promising prospect of MCC950 for the treatment of VMC as a specific inhibitor of the NLRP3 inflammasome activation.

### 7.4 Colchicine

Colchicine has been reported to inhibit the NLRP3 inflammasome activation *via* blocking microtubule assembly (Misawa et al., 2013). Pappritz et al. have reported that in C57BL6/j mice with CVB3-induced VMC, colchicine applied in the early stage of CVB3 infection could inhibit the splenic NLRP3 inflammasome activity, accompanied by alleviated immune cell infiltration, decreased cardiac troponin-1, and improved left ventricular function (Pappritz et al., 2022). These results could be explained by the fact that colchicine inhibited the infiltration of NLRP3-active inflammatory cells from the spleen to the heart (Pappritz et al., 2022). Furthermore, *in vitro* experiments demonstrated that colchicine suppressed the NLRP3 inflammasome activity in CVB3-infected HL-1 cardiomyocytes and fibroblasts, inhibiting their pro-inflammatory and pro-fibrotic capacities respectively (Pappritz et al., 2022). Importantly, colchicine applied to the CVB3-infected mice showed no exacerbation of CVB3 load, indicating that the colchicine might have little negative impact on anti-viral reactions of host cells and could be applied during viral persistence (Pappritz et al., 2022). However, previous research reported that colchicine injection could up-regulate the level of cardiac CVB3 mRNA and exacerbate CVB3-induced myocarditis, resulting in severe discomfort and higher mortality 3 days post-infection in CH3 mice (Smilde et al., 2016), which might partially be explained by the sensitivity difference between the two strains of mice, but indicated the potential cytotoxicity of colchicine. In summary, colchicine showed promising potential for balancing the modulation of excessive inflammation and the clearance of viruses. However, clinical studies, including follow-up biopsies to quantify myocardial inflammation and viral activity, are needed to evaluate the safety and efficacy of colchicine in human settings.

### 7.5 Interleukin-37

Interleukin-37 (IL-37), a member of the IL-1 cytokine family, has been demonstrated to alleviate CVB3-induced VMC in mice by suppressing the NF-κB pathway activation and inhibiting the NLRP3 inflammasome activity (Sun et al., 2022). In the well-constructed CVB3-induced VMC model of mice, IL-37 injection improved cardiac functions, manifesting as significantly higher LVEF, LVFS, IVSs and IVSd and significantly reduced the level of cardiac troponin I. Furthermore, the IL−37 treatment alleviated CVB3−induced inflammatory cell infiltration and fibrosis deposition in myocardium (Sun et al., 2022), which indicated the potential of IL-37 in ameliorating chronic inflammation and cardiac fibrosis. Though IL-37 has shown protective effects in CVB3-induced VMC, the mechanisms and possible side effects of IL-37 in treating VMC remain to be elucidated. More animal experiments are needed to confirm its safety and efficacy before admitting clinical application.

### 7.6 Interferons

Interferons (IFNs) are a class of broad-spectrum antiviral agents that can limit the proliferation of many viruses in the human body and treat the diseases caused by virus infection. IFN-α and IFN-β exert antiviral effects mainly through IFN-stimulated genes (ISGs). ISGs respond to IFN-α and IFN-β produced upon infection, thus initiating the antiviral state in bystander cells. The main mechanisms involve myxovirus resistance 1 (MX1), IFN-inducible double-stranded RNA-dependent protein kinase (PKR; encoded by EIF2AK2), 2′-5′-oligoadenylate synthetase (OAS), etc (McNab et al., 2015).

IFNs have long been used in the treatment of VMC, and their effectiveness has been demonstrated in several clinical trials (Mirić et al., 1995; Kühl et al., 2003) and case reports (Daliento et al., 2003; Canales Siguero et al., 2021). In the past, people often attributed the effect of IFN treatment on VMC to its broad-spectrum antiviral activity. However, recent studies have shown complex interactions between IFNs and the NLRP3 inflammasome. IFNs inhibit the NLRP3 inflammasome activation by several mechanisms involving the phosphorylation of STAT1 transcription factor and the promotion of IL-10 transcription (Guarda et al., 2011), thus alleviating the inflammatory responses caused by the virus and its subsequent adverse symptoms. In addition, some experiments have shown that IFN-γ could rescue mice with chronic VMC by reducing IL-1β levels. In another experiment, IFN-γ-deficient and wild-type BALB/c mice were inoculated with CVB3 on day 0. Hearts were collected for analysis on day 35, and a dramatic increase in IL-1β level in IFN-γ-deficient mice was observed (Fairweather et al., 2004). This suggests that IFN-γ may achieve the purpose of alleviating VMC by inhibiting the NLRP3 inflammasome activity. The results above indicate that part of the demonstrated effectiveness of IFNs in treating VMC is through the NLRP3 inflammasome pathway.

From what we have discussed above, a variety of drugs suppressing the NLRP3 inflammasome activation or inhibiting the IL-1β activity may be potential therapies for VMC, while the safety and efficacy of some drugs need further elucidation by more comprehensive animal experiments and clinical trials. Apart from chemical drugs targeting the NLRP3 inflammasome-IL-1β axis, NLRP3 knockout might also be a potential therapy for VMC. However, Wang et al. demonstrated that NLRP3 knockout in CVB3-infected mice exacerbated viral infection, cardiac injury, and cardiac dysfunction (Wang et al., 2019), which indicated the potential risks of NLRP3 knockout in treating VMC. More research is needed to assess the safety and efficacy of NLRP3 knockout as a therapy for VMC.

## 8 Conclusion

At present, the roles of inflammasomes, mainly the NLRP3 inflammasome, in VMC are well established. During the infectious phase, the NLRP3 inflammasome is activated, induces the release of IL-1β and IL-18 and promotes pyroptosis, participating significantly in the development of VMC. Based on the inflammasome activation pathways, we proposed possible therapies. Although the efficacy and safety of some therapies remain to be further tested, we believe that therapies targeting the inflammasome pathways are promising in treating VMC.

Nowadays, the interaction between SARS-Cov-2 and myocarditis has received a lot of attention. Myocarditis is regarded as a rare complication of COVID-19, and the pathogenic process of COVID-19-related myocarditis also indicates the involvement of the NLRP3 inflammasome. However, there is a lack of direct evidence on the role of the NLRP3 inflammasome in COVID-19-related myocarditis, and further research is needed to better understand COVID-19-related myocarditis.

## Author contributions

JX, ZZ and YZ were in charge of searching all the relative papers and writing this manuscript. HL, KH and SY gave their valuable and professional suggestions and guide in organizing and drafting this manuscript. All authors contributed to the article and approved the submitted version.
